# National health insurance and surgical care for injured people, Ghana

**DOI:** 10.2471/BLT.20.255315

**Published:** 2020-09-28

**Authors:** Adam Gyedu, Stephanie K Goodman, Micah Katz, Robert Quansah, Barclay T Stewart, Peter Donkor, Charles Mock

**Affiliations:** a Department of Surgery, KNUST School of Medicine and Dentistry, Kwame Nkrumah University of Science and Technology, Postal Mail Bag, University Campus, Kumasi, Ghana.; b Stanford Children’s Health, Palo Alto, United States of America (USA).; c Department of Surgery, University of Utah, Salt Lake City, USA.; d Department of Surgery, University of Washington, Seattle, USA.

## Abstract

**Objective:**

To determine the association between having government health insurance and the timeliness and outcome of care, and catastrophic health expenditure in injured patients requiring surgery at a tertiary hospital in Ghana.

**Methods:**

We reviewed the medical records of injured patients who required surgery at Komfo Anokye Teaching Hospital in 2015–2016 and extracted data on sociodemographic and injury characteristics, outcomes and out-of-pocket payments. We defined catastrophic health expenditure as ≥ 10% of the ratio of patients’ out-of-pocket payments to household annual income. We used multivariable regression analyses to assess the association between having insurance through the national health insurance scheme compared with no insurance and time to surgery, in-hospital mortality and experience of catastrophic health expenditure, adjusted for potentially confounding variables.

**Findings:**

Of 1396 patients included in our study, 834 (60%) were insured through the national health insurance scheme. Time to surgery and mortality were not statistically different between insured and uninsured patients. Insured patients made smaller median out-of-pocket payments (309 United States dollars, US$) than uninsured patients (US$ 503; *P* < 0.001). Overall, 45% (443/993) of patients faced catastrophic health expenditure. A smaller proportion of insured patients (33%, 184/558) experienced catastrophic health expenditure than uninsured patients (60%, 259/435; *P* < 0.001). Insurance through the national health insurance scheme reduced the likelihood of catastrophic health expenditure (adjusted odds ratio: 0.27; 95% confidence interval: 0.20 to 0.35).

**Conclusion:**

The national health insurance scheme needs strengthening to provide better financial risk protection and improve quality of care for patients presenting with injuries that require surgery.

## Résumé

**Objectif:**

Déterminer s'il existe un lien entre le fait d'avoir une assurance maladie gouvernementale d'une part, et la rapidité et l'efficacité des soins de l'autre, mais aussi les dépenses de santé catastrophiques chez les patients blessés nécessitant une intervention chirurgicale dans un hôpital tertiaire au Ghana.

**Méthodes:**

Nous avons examiné les dossiers médicaux des blessés devant faire l'objet d'une intervention chirurgicale à l'hôpital universitaire Komfo Anokye en 2015–2016, et nous en avons extrait des données sur les caractéristiques sociodémographiques et les particularités des lésions traitées, les résultats et les paiements à charge des patients. Nous avons considéré que les dépenses de santé catastrophiques équivalaient à une proportion ≥ 10% entre les paiements à charge des patients et les revenus annuels du ménage. Nous avons mené des analyses par régression multivariée afin d'évaluer les liens potentiels entre le fait d'être assuré via le régime national d'assurance maladie, ou de ne pas être assuré du tout, et le délai d'attente avant l'intervention chirurgicale, la mortalité hospitalière et les dépenses de santé catastrophiques, ajustées en fonction des potentielles variables confusionnelles.

**Résultats:**

Sur les 1396 patients figurant dans notre étude, 834 (60%) étaient assurés via le régime national d'assurance maladie. Le délai d'attente avant l'intervention chirurgicale et la mortalité ne présentaient aucune différence sur le plan statistique entre patients assurés et non assurés. Les patients assurés devaient généralement débourser moins (309 dollars américains) que les patients non assurés (503 dollars américains; *P* < 0,001). Au total, 45% (443/993) des patients ont dû faire face à des dépenses de santé catastrophiques. Une plus faible quantité de patients assurés (33%, 184/558) que de patients non assurés (60%, 259/435; *P* < 0,001) y ont été confrontés. Le fait d'être assuré via le régime national d'assurance maladie réduisait la probabilité de devoir assumer des dépenses de santé catastrophiques (odds ratio ajusté: 0,27; intervalle de confiance de 95%: 0,20 à 0,35).

**Conclusion:**

Le régime national d'assurance maladie doit être renforcé afin d'offrir une meilleure protection contre les risques financiers, et d'améliorer la qualité des soins pour les patients présentant des lésions qui nécessitent une intervention chirurgicale.

## Resumen

**Objetivo:**

Determinar la asociación entre disponer de un seguro médico del gobierno y la puntualidad y el resultado de la atención, y el gasto catastrófico en salud de los heridos que necesitan una intervención quirúrgica en un hospital terciario de Ghana.

**Métodos:**

Se analizaron las historias clínicas de los heridos a quienes se les practicó una intervención quirúrgica en el Hospital Universitario Komfo Anokye entre 2015 y 2016 y se obtuvieron los datos sobre las características sociodemográficas y de las lesiones, los resultados y los pagos de bolsillo. Se definió el gasto catastrófico en salud como ≥ 10 % del cociente entre los pagos de bolsillo de los pacientes y los ingresos anuales por hogar. Se aplicaron análisis de regresión multivariable para evaluar la asociación entre disponer de un seguro a través del plan nacional de seguro médico en comparación con no tener seguro y el tiempo hasta la intervención quirúrgica, la mortalidad intrahospitalaria y la experiencia de los gastos catastróficos en salud, todo esto ajustado a las variables que pudieran dar lugar a confusión.

**Resultados:**

De los 1396 pacientes que se incluyeron en este estudio, 834 (60 %) estaban asegurados a través del plan nacional de seguro de salud. El tiempo hasta la intervención quirúrgica y la mortalidad no fueron estadísticamente diferentes entre los pacientes asegurados y los no asegurados. Los pacientes asegurados efectuaron pagos de bolsillo en promedio menores (309 dólares de los EE. UU., USD) que los pacientes no asegurados (503 USD; *P* < 0,001). En total, el 45 % (443/993) de los pacientes registraron gastos catastróficos de salud. Un porcentaje menor de pacientes asegurados (33 %, 184/558) tuvo que pagar gastos catastróficos de salud que los pacientes no asegurados (60 %, 259/435; *P* < 0,001). El seguro a través del plan nacional de seguro médico redujo la probabilidad de que se incurriera en gastos catastróficos de salud (oportunidad relativa ajustada: 0,27; intervalo de confianza del 95 %: 0,20 a 0,35).

**Conclusión:**

Se debe fortalecer el plan nacional de seguro médico para ofrecer una mejor protección contra los riesgos financieros y mejorar la calidad de la atención a los pacientes con lesiones que requieran intervenciones quirúrgicas.

## ملخص

الغرض تحديد الارتباط بين الحصول على تأمين صحي حكومي، والتوقيت المناسب للرعاية ونتائجها، والنفقات الصحية الجائرة للمرضى المصابين الذين يحتاجون للجراحة في مستشفى فوق ثانوي في غانا.

الطريقة قمنا بمراجعة السجلات الطبية للمرضى المصابين الذين احتاجوا لجراحة في مستشفى كومفو أنوكي التعليمي في الفترة 2015-2016، واستخرجنا بيانات عن الجوانب الاجتماعية والسكانية، وخصائص الإصابة، والنتائج والمدفوعات الشخصية. وقمنا بتحديد النفقات الصحية الجائرة بأنها أكبر من أو تساوي 10% من نسبة المدفوعات الشخصية للمريض إلى الدخل السنوي للأسرة. لقد استخدمنا تحليلات التحوف متعدد المتغيرات لتقييم الارتباط بين الحصول على التأمين من خلال نظام التأمين الصحي الوطني، مقارنة بعدم وجود تأمين ووقت للجراحة، والوفيات داخل المستشفى، وتجربة الإنفاق الصحي الجائر، مع تعديلها لمتغيرات مربكة محتملة.

النتائج من بين 1396 مريضًا شملتهم الدراسة لدينا، تم تأمين 834 مريضًا (60%) من خلال نظام التأمين الصحي الوطني. لم يكن وقت الجراحة والوفيات مختلفين من الناحية الإحصائية بين المرضى المؤمَّن عليهم وغير المؤمَّن عليهم. قام المرضى المؤمَّن عليهم بدفع متوسط مدفوعات شخصية (309 دولار أمريكي) أقل من المرضى غير المؤمَّن عليهم (503 دولار أمريكي؛ نسبة الاحتمال أقل من 0.001). بشكل عام، تكبّد 45% (443/993) من المرضى نفقات صحية جائرة. تعرضت نسبة أقل من المرضى المؤمَّن عليهم (33%، 184/558) لنفقات صحية جائرة، بشكل أكثر من المرضى غير المؤمَّن عليهم (60%، 259/435؛ نسبة الاحتمال أقل من 0.001). قلل التأمين من خلال نظام التأمين الصحي الوطني من احتمال الإنفاق الصحي الجائر (نسبة الاحتمال المعدّلة: 0.27؛ فاصل الثقة 95%: 0.20 إلى 0.35).

الاستنتاج يحتاج نظام التأمين الصحي الوطني إلى الدعم حتى يوفر حماية أفضل للمخاطر المالية، ويُحسّن جودة رعاية المرضى الذين يعانون من إصابات تحتاج إلى الجراحة.

## 摘要

**目的:**

确定加纳三级医院需要手术的受伤患者中，享有政府医疗保险与护理及时性及结果，以及灾难性卫生支出之间的关联性。

**方法:**

我们回顾了 2015 至 2016 年间 Komfo Anokye 教学医院需要手术的受伤患者病历，并提取了社会人口统计，以及受伤特征、疗效和自费相关数据。灾难性卫生支出这里指患者自费部分占家庭年收入比例的10%。我们利用多变量回归分析来评估相较于没有保险，通过国民健康保险计划参保与手术时间、住院死亡率以及灾难性卫生支出之间的关联性，并针对可能的模糊变量进行调整。

**结果:**

1396 名接受调查的患者中，834 (60%) 名通过国民健康保险计划参保。手术时间和死亡率方面，参保和未参保之间没有明显差异。但自费部分，参保患者（309 美元）低于未参保患者（503 美元;*P* < 0.001）。总体而言，45% (443/993) 的患者面临灾难性卫生支出。参保患者 (33%, 184/558) 面临灾难性卫生支出的几率低于未参保患者 (60%, 259/435; *P* < 0.001)。通过国民健康保险计划参保降低了灾难性卫生支出的几率（调整后优势比：0.27；95％ 置信区间：0.20 至 0.35）。

**结论:**

需要加强国民健康保险计划，从而为需要手术的受伤患者提供更稳妥的财务风险保护并改善其疗效。

## Резюме

**Цель:**

Определить связь между наличием государственного медицинского страхования и своевременностью и результатами лечения, а также катастрофическими расходами на услуги здравоохранения для раненых, которым требуется хирургическое вмешательство в специализированной больнице в Гане.

**Методы:**

Авторы изучили медицинские карты раненых, которым требовалось хирургическое вмешательство в учебной клинике Komfo Anokye в период с 2015 по 2016 год, и получили данные о социально-демографических характеристиках и характеристиках травм, результатах лечения и собственных расходах. Авторы определили катастрофические расходы на услуги здравоохранения как ≥ 10% от соотношения собственных расходов пациента к годовому доходу семьи. Для оценки связи между наличием или отсутствием страховки в рамках государственной системы медицинского страхования и временем до операции, показателями внутрибольничной смертности и опытом катастрофических расходов на услуги здравоохранения с поправкой на потенциально искажающие переменные использовался многомерный регрессионный анализ.

**Результаты:**

Из 1396 пациентов, включенных в исследование, 834 (60%) были застрахованы в рамках государственной системы медицинского страхования. Время до операции и показатели смертности статистически не различались между застрахованными и незастрахованными пациентами. Собственные расходы застрахованных пациентов (309 долларов США) в среднем были меньше, чем собственные расходы незастрахованных пациентов (503 доллара США; *P* < 0,001). В целом 45% пациентов (443/993) столкнулись с катастрофическими расходами на услуги здравоохранения. Застрахованные пациенты (33%, 184/558) понесли меньше катастрофических расходов на услуги здравоохранения, чем незастрахованные пациенты (60%, 259/435; *P* < 0,001). Страхование в рамках государственной системы медицинского страхования снизило вероятность катастрофических расходов на услуги здравоохранения (скорректированное отношение шансов: 0,27; 95%-й ДИ: от 0,20 до 0,35).

**Вывод:**

Следует укреплять государственную систему медицинского страхования, чтобы обеспечить лучшую защиту от финансовых рисков и повысить качество обслуживания раненых, требующих хирургического вмешательства.

## Introduction

Trauma is a leading cause of morbidity and mortality worldwide.^1^ Low- and middle-income countries bear a disproportionate share of this burden.^1^ Improved trauma care may prevent up to 38% (1 965 000/5 130 000) of all injury deaths in low- and middle-income countries.^2^ Improvements in trauma care are possible in any environment with low-cost investments in planning and organization, human resources (skills, training and staffing) and physical resources (equipment and supplies).^3^ As countries work to build their capacity to provide care for people with injuries, it is not enough for care to be effective; it must also be affordable for the patients.^4^

Most injuries are unexpected emergencies. As a result, individuals and families do not have the opportunity to plan for the financial expenditure, which may make the economic burden of health-care costs and lost income from work especially serious. Although repeatedly demonstrated to be cost-effective in the long-run, the costs of diagnostic imaging, surgery, involvement of multiple specialists and rehabilitation add up to substantial costs of treatment in the short-term.^5^ Requirements for out-of-pocket payments during emergency care have been shown to slow service delivery,^6^ cause delays in surgical intervention, and contribute to preventable death and disability among injured patients.^7^

In addition to improving care, the effect of cost of care on families must be considered. Universal health coverage (UHC), government subsidies and insurance schemes are intended to limit catastrophic health expenditure, a term used by the World Bank, World Health Organization and the Lancet Commission on Global Surgery to characterize the financial impact of medical cost on people. These organizations have set a target of 100% financial protection from catastrophic health expenditure by 2030.^4^ Ghana established a national health insurance scheme in 2003 to expand access to medical care and increase its affordability. The scheme covers all emergencies and much of trauma care.^8^^,^^9^ However, some aspects of trauma care are not covered (e.g. many medications, advanced diagnostic imaging, prosthetics and rehabilitation, and mortuary services). Enrolment in the national health insurance scheme is voluntary at an annual fee of Ghanaian cedi (GH₵) 24 (4.2 United States dollars, US$, at the current rate of exchange). In 2013, 68% of Ghanaians were insured through the national health insurance scheme.^10^ However, because both the reimbursement rate to the health-care providers and timeliness of reimbursement have declined, resources needed for delivery of essential services have become unavailable at many health facilities.^8^^,^^11^^,^^12^ As a result, some hospitals pass the cost of essential care back to patients.

Here, we aimed to determine the association between insurance status and the timeliness of surgery and mortality in patients who presented to a tertiary hospital in Ghana with a serious traumatic injury. We also determined the proportion of these patients who experienced catastrophic health expenditure as a result of the care they received for the injury and if this expenditure was associated with insurance status.

## Methods

### Setting

Ghana is a lower middle-income country with a population of 30 million.^13^ Komfo Anokye Teaching Hospital is the second largest of five teaching hospitals in Ghana, and is the main referral centre for the middle third of the country, serving about 10 million people. The hospital has 1000 beds and 34 surgeons, and receives 2000 trauma admissions a year.^14^ It offers the range of trauma care including emergency, critical and acute care, surgery, reconstruction and rehabilitation.^15^^–^^18^

### Study design

We undertook a retrospective chart review of patients who presented to the Komfo Anokye Teaching Hospital accident and emergency centre with a traumatic injury needing surgery at the time of presentation. We reviewed the paper records of all such patients who presented from 1 January 2015 to 31 December 2016. We excluded patients with private health insurance because it provides better resources for these patients. We extracted data on: patient age, sex, occupation, cause of injury, severity of injury, vital signs at presentation, insurance status (through the national health insurance scheme or uninsured), patient outcome (died or alive at discharge), and out-of-pocket payments made. We determined patients’ alertness at presentation and severity of injury using the South African Triage Scale^19^ and Kampala Trauma Score II,^20^ respectively. We determined the need for surgery from the notes of the surgery team that assessed the patient in the emergency department. We calculated the time to surgery from presentation and length of postoperative hospital stay from the date and time stamps recorded in patient records. We determined whether the patient experienced a delay in surgery because of financial reasons if there was explicit information in the chart noting that surgery was delayed because the patient could not promptly make out-of-pocket payments for certain services (e.g. laboratory tests, diagnostic imaging, blood products, medications and surgery).

### Data analysis

#### Time to surgery and death

We did a bivariate analysis to assess the association between patients’ insurance status and several covariates that can affect outcome (age, blood pressure, respiratory rate, neurological status, cause of injury, number of serious injuries, site(s) of injuries and patient’s annual income) and that are often distributed differently in groups of trauma patients. We also did multivariable regression analyses with complete case analysis (only including participants with no missing data on the variables of interest) to assess the association between insurance status and the primary outcomes (time to surgery and in-hospital death) adjusted for covariates that were significant at *P* < 0.10 in the bivariate analysis. We used linear regression analysis to assess time to surgery and logistic regression analysis to assess mortality.

#### Financial risk

To determine the proportion of patients who experienced catastrophic health expenditure, we excluded patients whose records had no information on occupation and patients with missing information on out-of-pocket payments. We also excluded children (< 15 years) and non-working students since they did not have individual incomes. We included patients who were unemployed if this status was recorded in their chart, and classed them as having no individual annual income.

We used two different methods to determine catastrophic health expenditure based on individual and household annual income. First, we grouped individual patients according to occupation, as recorded in the medical chart, based on the *International standard classification of occupations*.^21^ We then used the Ghana *Living standards survey: labour force report* to determine the patients’ average annual incomes based on their occupation group and sex, and adjusted for inflation according to their year of admission (2015 or 2016).^22^^,^^23^ We determined the out-of-pocket payments made by patients from hospital billing records on discharge from the hospital. The ratio of out-of-pocket payments to annual income at the individual level for each patient was calculated as: individual patient’s out-of-pocket payments/individual patient’s annual income. Second, we extracted mean annual household income data from the Ghana *Living standards survey: main report*.^10^ The reported mean annual household income was (US$ 4499, US$ 1 = GH₵ 3.7 at the time of the study);^24^ the first to fifth quintile annual household incomes were US$ 1776, US$ 2891, US$ 4006, US$ 4570, and US$ 6811, respectively. We grouped patients into quintiles based on their individual annual incomes. Each quintile was then assigned the likely annual household income for that quintile based on the means from the Ghana *Living standards survey*, adjusted for inflation. We calculated the ratio of out-of-pocket payments to annual income at the household level for each patient as: individual patient’s out-of-pocket payments divided by patient’s assigned annual household income.

We calculated the proportion of patients who presented with injuries that required immediate surgery and who experienced catastrophic health expenditure using the previously published thresholds of 5%, 10%, 15%, and 20% of the household annual income.^25^^–^^28^ We made these calculations at both the individual and household level. We used the 10% threshold at the household level, which is the most commonly used threshold for estimating catastrophic health expenditure, as the outcome variable. We did bivariate and multivariable logistic regression analyses with the covariates selected as explained earlier.

We used Stata v14 (StataCorp. LP, College Station, United States of America) for all analyses.

### Ethics

The Kwame Nkrumah University of Science and Technology Committee for Human Research and Publication Ethics approved the study (Protocol CHRPE/AP/467/17).

## Results

### Study sample

From January 2015 to December 2016, 1408 patients presented to Komfo Anokye Teaching Hospital accident and emergency centre with injuries that required surgery at the time of presentation. We excluded 12 (1%) patients because they had private insurance, which left 1396 for the analysis. Most patients (834 patients; 60%) were insured through the national health insurance scheme. The insured group had fewer males and fewer people in the 15–55-year age group (Table 1). There were no significant differences between insured and uninsured patients for: cause of injury, site of serious injuries, number of serious injuries, acuity level in the South African Triage Scale and Kampala Trauma Score II scores at triage. Time to surgery, duration of surgery and in-hospital mortality were also similar between the two groups. Although there was no statistically significant difference in time to surgery between the groups, only 6% (53/834) of patients insured through the national health insurance scheme experienced delays for financial reasons as recorded in the medical charts compared with 17% (97/562) of uninsured patients (*P* < 0.001). Patients with insurance had a longer postoperative hospital stay (median: 15 days; interquartile range, IQR: 8 to 28) compared with uninsured patients (median: 12 days; IQR: 7 to 22; *P* = 0.03). We identified a subset of operations that should have started within 8 hours of admission (e.g. operations for bleeding or care of open wounds). Most of these urgent operations were started after an inappropriately long delay (> 8 hours), with no statistically significant difference between the insured and uninsured groups (Table 1).

**Table 1:** Characteristics and outcomes of patients presenting with injuries requiring surgery, Komfo Anokye Teaching Hospital, Ghana, 2015–2016

Variable	Insured^a^ (*n* = 834)	Uninsured (*n* = 562)	*P*
**Characteristics **			
Sex, no. (%)			
Male	554 (66)	402 (72)	0.04
Female	280 (34)	159 (28)	
Missing	0 (0)	1 (0.2)	
Age in years, no. (%)			
< 5	30 (4)	12 (2)	< 0.001
5–14	109 (13)	45 (8)	
15–55	534 (64)	422 (75)	
> 55	161 (19)	82 (15)	
Missing	0 (0)	1 (0.2)	
Systolic blood pressure at triage in mmHg, no. (%)			
> 89	719 (86)	512 (91)	0.944
50–89	23 (3)	16 (3)	
Missing	92 (11)	34 (6)	
Respiratory rate at triage in breaths/minute, no. (%)			
10–29	818 (98)	549 (98)	0.475
> 30	12 (1)	8 (1)	
< 9	0 (0)	1 (0.2)	
Missing	4 (1)	4 (1)	
Neurological status at triage, no. (%)			
Alert	786 (94)	523 (93)	0.450
Responds to verbal stimuli	26 (3)	22 (4)	
Responds to painful stimuli	17 (2)	15 (3)	
Unresponsive	5 (1)	1 (0.2)	
Missing	0 (0)	1 (0.2)	
Cause of injury, no. (%)			
Road traffic crash	468 (56)	314 (56)	0.294
Fall	204 (24)	118 (21)	
Blunt trauma	56 (7)	42 (7)	
Animal bite	54 (6)	51 (9)	
Burn	12 (1)	4 (1)	
Gunshot	23 (3)	20 (4)	
Other	14 (2)	9 (2)	
Missing	3 (0.4)	4 (1)	
Site of serious injuries, no. (%)^b^			
Head or neck	176 (21)	111 (20)	0.551
Chest	30 (4)	18 (3)	0.692
Spine	23 (3)	10 (2)	0.238
Abdomen or pelvis	29 (4)	23 (4)	0.552
Extremity	759 (91)	521 (93)	0.270
Number of serious injuries, no. (%)			
Multiple	148 (18)	106 (19)	0.596
Single	686 (82)	456 (81)	
Triage acuity level, no. (%)^c,d^			
Green	25 (3)	14 (2)	0.540
Yellow	434 (52)	271 (48)	
Orange	293 (35)	214 (38)	
Red	44 (5)	32 (6)	
Missing	38 (5)	31 (6)	
Kampala Trauma Score II, no. (%)			
Mild (9–10)	443 (53)	321 (57)	0.874
Moderate (7–8)	280 (34)	191 (34)	
Severe (≤ 6)	19 (2)	14 (2)	
Missing	92 (11)	36 (6)	
Individual annual income in US$, median (IQR)	2502 (1696 to 3362)	2695 (1696 to 3362)	0.99
Experienced financial delay, no. (%)	53 (6)	97 (17)	< 0.001
Out-of-pocket payment in US$, median (IQR)^e^	309 (181 to 521)	503 (298 to 759)	< 0.001
Out-of-pocket payment to individual annual income ratio, median (IQR)^f^	0.14 (0.07 to 0.24)	0.21 (0.13 to 0.33)	0.0003
Time to surgery in hours, median (IQR)	50 (21 to 167)	42 (18 to 143)	0.611
Overly long time to surgery (> 8 hours) for urgent cases, no. (%)^g^	398/458 (86.9)	246/298 (82.6)	0.10
Duration of surgery in hours, median (IQR)	1.3 (0.8 to 1.9)	1.4 (0.9 to 1.9)	0.13
Length of postoperative stay in days, median (IQR)	15 (8 to 28)	12 (7 to 22)	0.03
In-hospital mortality, no. (%)	22 (3)	10 (2)	0.29

IQR: interquartile range; SATS: South African Triage Scale; SD: standard deviation; US$: United States dollar.

^a^ Insured through national health insurance scheme.

^b^ Total sums to > 1396 because some patients had injuries in more than one place.

^c^ According to South African Triage Scale.

^d^ Green: routine (needs to be seen within 4 hours); yellow: urgent (needs to be seen within 1 hour); orange: very urgent (needs to be seen within 10 minutes); and red: emergency (needs to be seen immediately).

^e^ Mean out-of-pocket payment was US$ 398 (SD 332) and US$ 610 (SD 778) for insured and uninsured patients, respectively.

^f^ Mean out-of-pocket payments to individual annual income ratio was 0.19 and 0.30 for insured and uninsured patients, respectively.

^g^ Urgent operations include those that should start within 8 hours of admission, e.g. operations to stop bleeding or for open wounds. There were 458 such urgent operations for insured patients and 298 for uninsured patients.

Notes: US$ 1 = 3.7 Ghanaian cedi at the time of the study. The *χ*^2^ test for categorical variables and *t*-test for continuous variables were used to assess differences between insured and uninsured patients.

### Time to surgery and death

In the multivariable analyses, time to surgery was not statistically different between patients insured through the national health insurance scheme and uninsured patients: adjusted *β* = 5.36 hours (95% confidence interval, CI: −15.02 to 25.74; Table 2). Similarly, mortality was not significantly different between insured and uninsured patients (adjusted odds ratio, aOR: 1.55; 95% CI: 0.72 to 3.30; Table 3).

**Table 2:** Factors associated with time to surgery in patients presenting with injuries requiring surgery, Komfo Anokye Teaching Hospital, Ghana, 2015–2016

Variable	Crude *β* (95% CI)	Adjusted *β* (95% CI)
**Insurance status**		
Uninsured	Reference	Reference
Insured^a^	5.32 (−15.21 to 25.86)	5.36 (−15.02 to 25.74)
**Sex**		
Male	Reference	Reference
Female	34.20 (12.95 to 55.45)	26.61 (4.73 to 48.49)
**Age**	0.82 (0.35 to 1.29)	0.67 (0.19 to 1.16)

CI: confidence interval.

^a^ Through the national health insurance scheme.

Notes: We included 1257 patients in the model. Residuals from the model had normal distribution.

**Table 3:** Factors associated with death in patients presenting with injuries requiring surgery, Komfo Anokye Teaching Hospital, Ghana, 2015–2016

Variable	Crude OR (95% CI)	Adjusted OR (95% CI)
**Insurance status**		
Uninsured	Reference	Reference
Insured^a^	1.50 (0.70 to 3.18)	1.55 (0.72 to 3.30)
**Sex**		
Male	Reference	Reference
Female	0.60 (0.26 to 1.41)	0.39 (0.16 to 0.99)
**Age**	1.02 (1.00 to 1.03)	1.03 (1.01 to 1.04)

OR: odds ratio; CI: confidence interval.

^a^ Through the national health insurance scheme.

Notes: We included 1395 patients in the model. Residuals from the model had normal distribution.

### Financial risk

We excluded 403 patients (29% of all patients) from the analysis of catastrophic health expenditure because they did not have data to evaluate financial risk protection, either because of missing out-of-pocket payments or missing income: 85 lacked information on occupation, 11 lacked information on out-of-pocket payment and 307 were children or non-working students. Thus, we included 993 patients in this analysis. Of these patients, 672 were male (68%), the median age was 38 years (IQR: 29 to 52) and 558 (56%) were insured through the national health insurance scheme. Most of these patients (963; 97%) had mild or moderate scores on the Kampala Trauma Score II. Only 58 (6%) patients were triaged red (i.e. needed to be seen immediately) with the South African Triage Scale. The median individual annual income and assigned annual household income of these 993 patients were US$ 2608 (IQR: 1696 to 3362) and US$ 6058 (IQR: 4372 to 7118), respectively. There was no significant difference in individual annual incomes of insured patients and uninsured patients (*P* = 0.99). Median out-of-pocket payments made by insured and uninsured patients were significantly different: US$ 309 (IQR: 181 to 521) and US$ 503 (IQR: 298 to 759), respectively (*P* < 0.001). Patients insured through the national health insurance scheme spent a smaller percentage of their annual income on out-of-pocket payments for this one episode of care for their injury than uninsured patients: median 14% (IQR: 7 to 24) and 21% (IQR: 13 to 33), respectively; *P* = 0.0003 (Table 1).

**Fig. 1:**
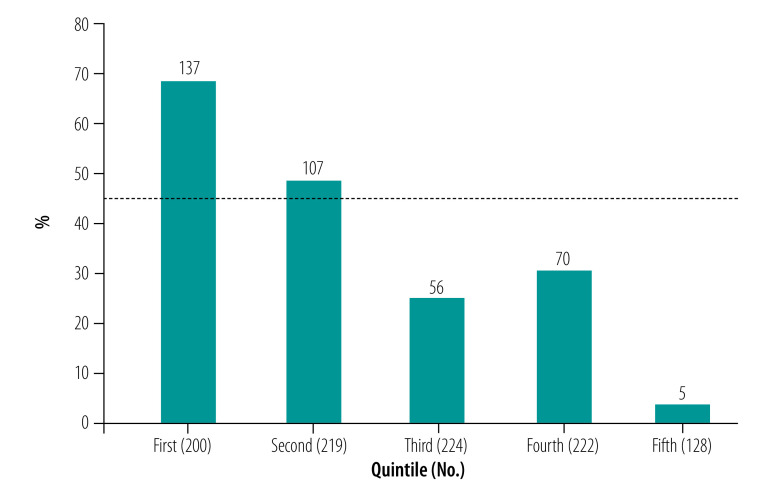
Percentage of patients presenting with injuries requiring surgery who experienced catastrophic health expenditure, by income quintile, Komfo Anokye Teaching Hospital, Ghana, 2015–2016

Notes: Based on a 10% threshold for catastrophic health expenditure for ratio of out-of-pocket expenditure to assigned annual household income. The dotted line represents the average rate of catastrophic health expenditure of 45%.

Table 4 shows the percentage of patients who experienced catastrophic health expenditure at different thresholds according to insurance status. Using the individual patient’s estimated annual income as the denominator, 55 to 92% (549/993 to 915/993) of patients experienced catastrophic health expenditure at various thresholds. With the patient’s assigned household income as the denominator, 21 to 72% (212/993 to 719/993) of patients faced catastrophic health expenditure at the same thresholds. Overall, for all thresholds considered, a smaller proportion of insured patients (33%; 184/558) experienced catastrophic health expenditure than uninsured patients (60%; 259/435; *P* < 0.001). A greater percentage of patients in the first (poorest) and second quintile of annual household income faced catastrophic health expenditure than patients in the third to fifth quintiles at the 10% threshold (*P* < 0.001; Fig. 1).

**Table 4:** Patients presenting with injuries requiring surgery who experienced catastrophic health expenditure, by insurance status, Komfo Anokye Teaching Hospital, Ghana, 2015–2016

Ratio of patient’s out-of-pocket expenditure to:	No. (%) of patients with catastrophic health expenditure			
	Threshold for catastrophic health expenditure			
	5%	10%	15%	20%
**Patient’s annual income**				
Insured^a^ (*n* = 558)	496 (89)	388 (70)	318 (57)	256 (46)
Uninsured (*n* = 435)	419 (97)	390 (90)	340 (78)	293 (67)
All patients (*n* = 993)	915 (92)	778 (78)	658 (66)	549 (55)
*P*	< 0.001	< 0.001	< 0.001	< 0.001
**Patient’s assigned annual household income**				
Insured^a^ (*n* = 558)	347 (62)	184 (33)	106 (19)	80 (14)
Uninsured (*n* = 435)	372 (86)	259 (60)	168 (39)	132 (30)
All patients (*n* = 993)	719 (72)	443 (45)	274 (28)	212 (21)
*P*	< 0.001	< 0.001	< 0.001	< 0.001

^a^ Through the national health insurance scheme.

Note: Catastrophic health expenditure was based on the ratio of out-of-pocket expenses to either patient’s annual income or assigned annual household income.

In multivariable analysis, having insurance through the national health insurance scheme reduced the odds of experiencing catastrophic health expenditure by 70% compared with having no insurance (aOR: 0.27; 95% CI: 0.20 to 0.35). Women had more than twice the odds of experiencing catastrophic health expenditure than men (aOR: 2.41; 95% CI: 1.77 to 3.28; Table 5).

**Table 5:** Factors associated with catastrophic health expenditure in patients presenting with injuries requiring surgery, Komfo Anokye Teaching Hospital, Ghana, 2015–2016

Variable	Crude OR (95% CI)	Adjusted OR (95% CI)
**Insurance status**		
Uninsured	Reference	Reference
Insured^a^	0.33 (0.26 to 0.43)	0.27 (0.20 to 0.35)
**Sex**		
Male	Reference	Reference
Female	2.36 (1.80 to 3.09)	2.41 (1.77 to 3.28)
**Age**	1.02 (1.01 to 1.02)	1.01 (1.01 to 1.02)

OR: odds ratio; CI: confidence interval.

^a^ Through the national health insurance scheme.

Notes: Based on a 10% threshold for catastrophic health expenditure for ratio of patients’ out-of-pocket expenditure to annual household income. We included 991 patients in the model. Residuals from the model had normal distribution.

Included in our 1396 patients were 119 (9%) patients who refused the recommended surgical intervention because of concerns about the likely out-of-pocket cost of treatment. Of these patients, the median out-of-pocket payment was US$ 148 (IQR: 102 to 215). Of the patients who refused the recommended surgical intervention, 67% (80/119) were uninsured compared with 33% (39/119) who were insured (*P* < 0.001).

### Sensitivity analysis

Income was not significantly different between the insured and uninsured groups (Table 1) and so we did not include this variable in the main multivariable analyses. We conducted a sensitivity analysis in which we added individual income to the multivariable analyses. This addition did not change the relationship between having insurance through the national health insurance scheme and any of the outcome variables (details are in the data repository).^29^

## Discussion

The national health insurance scheme was established to facilitate equitable and universal access to health care that protects all Ghanaians “against the need to pay out of pocket at the point of service.”^30^ Here, we found that having insurance was associated with fewer delays in care for financial reasons, as recorded in the medical charts, but the timeliness of surgery and mortality were similar for insured and uninsured patients. Having insurance through the national health insurance scheme reduced catastrophic health expenditure by 70%, but did not eliminate out-of-pocket payments. A large proportion of patients still faced catastrophic health expenditure, particularly women.

Timeliness of care is an important feature of service delivery and a useful focus for improvement in emergency and trauma care.^31^ For injured patients arriving at the emergency department who need a surgical intervention, care usually involves patient triage, resuscitation, assessments by multiple teams, performance of laboratory and imaging tests, preparation of the theatre and use of consumables. All these contribute to the cost of care that the national health insurance scheme is intended to cover. A recent review by a panel of physicians of preventable deaths in injured patients at Komfo Anokye Teaching Hospital reported that 50% (18/36) of deaths were either definitely preventable or possibly preventable.^7^ The panel further reported that delay in surgical intervention was responsible for 22% (9/41) of inappropriate care episodes that led to preventable deaths. The similar outcome we observe in the timeliness of surgical intervention between insured and uninsured might be because care was adequate for everyone and that lack of insurance did not disadvantage patients. However, all of the operations were identified as necessary at the time of admission and should have been done promptly; therefore, the median delays of 42 and 50 hours for insured and uninsured patients, respectively, indicate some degree of suboptimal care. In addition, most very urgent procedures did not start until after 8 hours and this delay did not change with insurance status. These findings suggest that care was delayed for other reasons.

Delays are likely caused by other equally important variables that can affect the timeliness of delivery of surgical services (e.g. staffing and theatre shortages, communication gaps, logistical inefficiencies and lack of medications or blood products). Reducing inefficiencies in these areas may reveal an association between the national health insurance scheme and timeliness of care for injured patients. Furthermore, mortality was not lower in injured patients with insurance than patients without insurance, which could be linked to the fact that having insurance did not improve timeliness of care.

Insurance through the national health insurance scheme reduced the odds of experiencing catastrophic health expenditure by 70% at a 10% threshold for out-of-pocket payments to household income. Previous studies that have examined general medical expenses also found that the national health insurance scheme offered protection against out-of-pocket payments.^32^^–^^34^ Patients insured through the national health insurance scheme who underwent various surgical procedures at the study hospital were reported to be less likely than uninsured patients to face financial catastrophe as a result of their surgery.^35^ However, over half of those patients still faced financial catastrophe because of out-of-pocket payments, even with insurance through the national health insurance scheme.^35^ Although the national health insurance scheme provided protection against catastrophic health expenditure in our study, only 13/834 (2%) of our insured patients did not make any out-of-pocket payments.

Despite the early success of the national health insurance scheme, total rate of timely reimbursement to service providers has declined due to over-reliance on a narrow tax base (the formal economy, which does not include most of the population), a large informal work sector (who make the most use of the scheme but contribute little to its financing), and greater than expected use of health-care services.^11^^,^^12^^,^^36^ As a result, health facilities are often unable to purchase needed resources to ensure efficient health-care delivery.^37^ For out-of-stock drugs and consumables approved by national health insurance scheme and certain services not approved by the national health insurance scheme, patients are required to pay out-of-pocket regardless of their condition.^38^ The vision behind Ghana’s national health insurance scheme is to cover all expenses with the goal of achieving UHC.^9^ Our study showed that the national health insurance scheme contributed to decreasing out-of-pocket payments.^39^ Nonetheless, 33% (184/558) of patients insured through the scheme faced catastrophic health expenditure. Therefore, a more robust national health insurance scheme is needed that reliably covers all types of essential care, including trauma care, if the international goal for 100% protection is to be met.

Of concern, almost every 10th patient in our study declined the recommended surgical intervention and left the hospital because of their worry about the cost of treatment. Although more of these patients were uninsured, the group still included patients insured through the national health insurance scheme. Estimations show that 60% of Ghanaians would be unable to afford surgery without resorting to some form of hardship financing, such as borrowing or liquidation of assets, despite being insured through the national health insurance scheme.^35^^,^^40^ Women are more likely to be in this position as they have lower median individual income than men in Ghana.^10^ Ensuring that the national health insurance scheme covers essential services, including emergency and trauma care, is not only good for health and future productivity, but also for gender equity.

Our study has some limitations. First, our data included individual patients’ out-of-pocket payments for care received and their occupation. These data allowed us to estimate the ratio of out-of-pocket payments to annual income for the individual patient. However, we did not have information on actual annual household income for our patient population. Therefore, we assigned patients to published annual household income quintiles. While this is reasonable, we could not find any literature to support this method. This method may lead to bias, especially among younger people who might have lower paying jobs but come from wealthier households. Second, our catastrophic health expenditure percentages represent an underestimate since we did not capture information on non-medical costs such as the cost of travel and cost of loss of wages due to hospital admission. Third, we had missing data for some of the variables (between 0.07% and 9.9% missing data), which could bias our results. Fourth, our study was done in one hospital and so the results may not be generalizable to all hospitals in Ghana. Despite these limitations, our study provides useful information on the proportion of injured patients who experienced catastrophic health expenditure and the protection provided by the national health insurance scheme. Our study also highlights the need to consider other factors affecting care that are independent of the national health insurance scheme to understand the effect of the national health insurance scheme on timeliness of care and mortality in patients with emergency conditions and injury.
